# Complexity of Daily Physical Activity Is More Sensitive Than Conventional Metrics to Assess Functional Change in Younger Older Adults

**DOI:** 10.3390/s18072032

**Published:** 2018-06-25

**Authors:** Wei Zhang, Michael Schwenk, Sabato Mellone, Anisoara Paraschiv-Ionescu, Beatrix Vereijken, Mirjam Pijnappels, A. Stefanie Mikolaizak, Elisabeth Boulton, Nini H. Jonkman, Andrea B. Maier, Jochen Klenk, Jorunn Helbostad, Kristin Taraldsen, Kamiar Aminian

**Affiliations:** 1 Laboratory of Movement Analysis and Measurement, Ecole Polytechnique Federale de Lausanne, 1015 Lausanne, Switzerland; anisoara.ionescu@epfl.ch (A.P.-I.); kamiar.aminian@epfl.ch (K.A.); 2 Robert Bosch Foundation for Medical Research, 70376 Stuttgart, Germany; michael.schwenk@rbk.de (M.S.); stefanie.mikolaizak@rbk.de (A.S.M.); jochen.klenk@rbk.de (J.K.); 3 Network Aging Research, Heidelberg University, 69115 Heidelberg, Germany; 4 Department of Electrical, Electronic and Information Engineering, University of Bologna, 40136 Bologna, Italy; sabato.mellone@unibo.it; 5 Department of Neuromedicine and Movement Science, Norwegian University of Science and Technology, 7491 Trondheim, Norway; beatrix.vereijken@ntnu.no (B.V.); jorunn.helbostad@ntnu.no (J.H.); kristin.taraldsen@ntnu.no (K.T.); 6 Department of Human Movement Sciences, Vrije Universiteit Amsterdam, Amsterdam Movement Sciences, 1081BT Amsterdam, The Netherlands; m.pijnappels@vu.nl (M.P.); n.h.jonkman@vu.nl (N.H.J.); andrea.maier@mh.org.au (A.B.M.); 7 School of Health Sciences, Faculty of Medicine, Biology and Health, University of Manchester, and Manchester Academic Health Science Centre, Manchester M13 9PL, UK; elisabeth.boulton@manchester.ac.uk; 8 Department of Medicine and Aged Care, @AgeMelbourne, Royal Melbourne Hospital, University of Melbourne, Melbourne 3050, Australia; 9 Institute of Epidemiology and Medical Biometry, Ulm University, 89081 Ulm, Germany

**Keywords:** wearable sensors, multivariate analysis, longitudinal study, functional decline, exercise intervention

## Abstract

The emerging mHealth applications, incorporating wearable sensors, enables continuous monitoring of physical activity (PA). This study aimed at analyzing the relevance of a multivariate complexity metric in assessment of functional change in younger older adults. Thirty individuals (60–70 years old) participated in a 4-week home-based exercise intervention. The Community Balance and Mobility Scale (CBMS) was used for clinical assessment of the participants’ functional balance and mobility performance pre- and post- intervention. Accelerometers worn on the low back were used to register PA of one week before and in the third week of the intervention. Changes in conventional univariate PA metrics (percentage of walking and sedentary time, step counts, mean cadence) and complexity were compared to the change as measured by the CBMS. Statistical analyses (21 participants) showed significant rank correlation between the change as measured by complexity and CBMS (ρ = 0.47, *p* = 0.03). Smoothing the activity output improved the correlation (ρ = 0.58, *p* = 0.01). In contrast, change in univariate PA metrics did not show correlations. These findings demonstrate the high potential of the complexity metric being useful and more sensitive than conventional PA metrics for assessing functional changes in younger older adults.

## 1. Introduction

The aging process is often accompanied by functional decline and increased risk of chronic diseases [1]. However, functional ability varies between older adults, depending on individual health condition and lifestyles. Physical inactivity is one of the known risk factors that can lead to morbidity and mortality [2]. The transition from work to retirement, which often occurs between 60 and 70 years of age, can involve a significant change in structured daily activities, with physical activity declining. A study on the Dutch population found that retirement introduces a reduction in physical activity from work-related transportation that is not compensated for by an increase in sports participation or an increase in non-sports leisure-time physical activity [3]. Thus, this population is of particular importance for addressing maintenance of their functional status.

In recent years, mobile health (mHealth) applications, incorporating wearable sensing technologies with modern mobile communication devices, are emerging. From the early adoption in younger populations for fitness tracking in particular, mHealth has been continuously developing and diversifying its applications for different populations [1,4]. The scalability of mHealth technologies enables data collection in diverse geographic locations over prolonged time periods [5]. This provides a new perspective in studying physical activities in real life and allows new analytical tools to be developed to analyze and present the data.

An earlier systematic review on body-worn, accelerometer-based physical activity monitoring revealed the challenge of achieving consensus on the reporting and the interpretation of the measurements provided by various mHealth applications [6]. Furthermore, the review pointed out that energy expenditure, walking time, and total activity are most frequently reported and are comparable variables across studies. In addition, several variables, such as walking time, number of steps, and cadence are the most widely adopted variables in research [5,7,8] to characterize walking pattern, the most common daily physical activity across all age groups. Descriptive statistics (e.g., mean, maximum values) are applied to the above walking parameters for analysis. Detrended fluctuation analysis proposed by Hausdorff et al. is used to quantify the stride-to-stride variability in supervised walking tasks [9]. However, physical activity involves multiple components and has more than one dimension. Different types of activities in daily life, as well as the quantity and the quality (performance) of each activity jointly determine a person’s functional status. Thus, a variable that models a person’s physical activity behaviour based on these aspects is warranted.

Complexity analysis as introduced by Paraschiv-Ionescu et al. [10] aims at combining both the quantity and quality dimensions of multiple, commonly performed daily activities into one metric to describe physical activity behaviour. This complexity metric has demonstrated discriminative power to distinguish groups of patients suffering from chronic pain [11,12]. Besides analyzing accelerometry-derived activities, the metric has been validated also for analyzing activity behaviour of older adults using an application based on wearable pressure insoles [13].

While several cross-sectional studies exist for clinical validation of complexity metrics, the sensitivity of those metrics for detecting changes in physical function over time has not yet been determined. Therefore, we aimed to examine the ability of the aforementioned complexity metric to detect change in a longitudinal intervention study conducted in younger older adults in comparison to conventionally applied univariate physical activity metrics. Within this context, we further explored the impact of smoothing sensor-based physical activity data on the complexity metric.

## 2. Materials and Methods

### 2.1. Study Protocol

The study is part of the larger PreventIT project [1], developing and testing an ICT-based mHealth system that enables early identification of risk for age-related functional decline, and engenders behavioural change in younger older adults (aged 60–70 years) in order to adopt a healthy, active lifestyle.

Thirty participants, aged between 60 and 70 years, were recruited at three different sites, Trondheim (Norway), Amsterdam (the Netherlands), and Stuttgart (Germany), to participate in a 4-week pre-post pilot intervention study. All participants were instructed by an experienced physical therapist or exercise therapist to follow an adapted Lifestyle-integrated Functional Exercise (aLiFE) programme specifically developed for improving balance and strength and increasing physical activity in younger older adults [14]. aLiFE was taught during four weekly home visits, and the participants were asked to integrate the aLiFE activities into everyday routines. Pre and post intervention, the participants completed a balance and mobility assessment using the Community Balance and Mobility Scale (CBMS). The scale has been validated to capture high-level balance, gait, and mobility performances in healthy active younger older adults based on the quality of performing the tasks [15]. CBMS assessments were performed in the research hospital or university by trained assessors. In addition, daily physical activity (PA) of each participant was measured twice for one week with wearable sensors. The participants were instructed to wear an inertial sensor (DynaPort, MoveMonitor, McRoberts, The Hague, The Netherlands) at their lower back at the level of L5 using an elastic belt during the day and night. The sensor needed to be removed when showering or during any water activity and needed to be put back on afterwards. The sensor did not need to be recharged during the one week measurement. All sensors were collected at the end of the measurement and raw sensor data was downloaded for offline data analysis. The first measurement was prior to the start of the intervention period. The measurement was repeated during the third week of the pilot study to capture the change of daily activity patterns during the intervention.

### 2.2. Sensor Data Processing

The sensor consists of a 3D accelerometer with sampling frequency of 100 Hz. The recording start time of each sensor was registered on the device by manual insertion of a timestamp. A non-commercial activity classification software was used to extract quantitative as well as qualitative features of PA from raw sensor data. The software is an outcome of the FARSEEING EU project (FP7/2007–2013, grant agreement 288940). It has been applied in studies with dementia patients [16] and older people residing in independent-living retirement homes [17]. The software has been further developed based on two datasets of elderly subjects. The first one is the ADAPT dataset [18], where video recording was performed using ceiling-mounted cameras in lab settings and an action camera in free-living conditions. The second dataset is from the University of Auckland [17], where subjects performed both scripted and unscripted activities of daily living collected in a free-living environment. First, the algorithm estimates Metabolic Equivalents (METs); signals are filtered and processed as described in [19]. An interval is labelled as ‘sedentary’, if associated energy expenditure is below or equal to 1.5 MET [20]. Otherwise, the interval is labelled as ‘active’. ‘Sedentary’ intervals with a mean angle between the vertical axis and the medio-lateral or the anterior–posterior direction of the trunk below 30° are labelled as ‘lying’. The ‘active’ intervals, where steps are detected are labelled as ‘walking’. Step detection is based on [21]. Each interval is then characterized by the category (label), the duration, and the activity counts (counts/minute) from which METs are estimated [19]. In addition, number of steps and the cadence (steps/minute) are extracted for each ‘walking’ bout. Data is labelled as ‘non-wearing’, if the sensor is detected lying flat with very low variance in acceleration signals for longer than half an hour.

**Figure 1:**
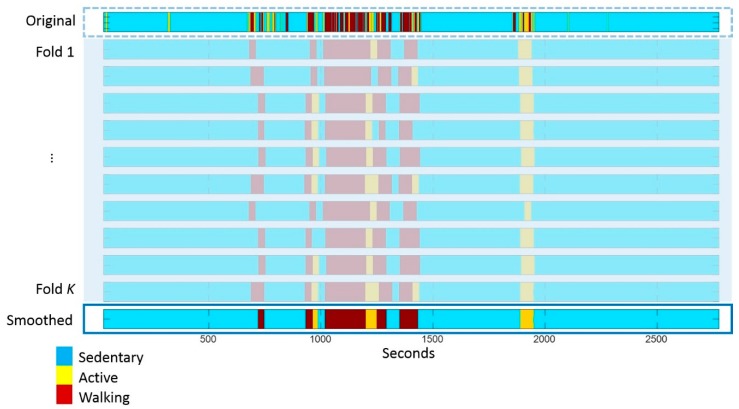
Smoothing activity classification output to aggregate activity bouts in a measurement time series of 45 min. The top bar shows the original PA sequence and the bottom bar shows the smoothed sequence.

The classified activities were sorted into natural days based on the registered timestamp. Days with less than 16 h of measurements (i.e., the first and the last day of the measurement) were excluded from further processing and analysis. Given the high resolution (1 s) of the PA output data, bouts of one activity may be interspersed with short episodes of other activities. For example, short breaks of a few seconds are often present during one walking bout due to environmental factors (e.g., walking episodes whiles shopping in a supermarket), which may lead to a string of several walking episodes rather than one continuous walking bout. Such short breaks during an activity introduce artificial changes in the dynamics of the PA time series, which are not relevant to one’s physical behaviour. Therefore, a smoothing technique was devised to filter such artefacts and to aggregate bouts in the original PA output belonging to the same PA category based on the following steps, as illustrated in Figure 1. First, we applied a moving forward sliding window of 30 s without overlap to smooth the PA time series [22]. The activity category of these 30 s was replaced by the activity with the highest density (counts) within the window. Second, the process was repeated for K folds. At each fold, the smoothing starts with a random shift between 1 and 30 s at the beginning of the time series. Third, the activity category of each second in the aggregated time series was determined by the majority vote of the K-fold smoothing. The sliding window length was chosen according to the ‘barcode’ design (explained in the later section Complexity analysis), where 30-s is the threshold of activity duration corresponding to indoor walking.

### 2.3. Univariate Analysis

For each activity bout, its duration and activity counts per minute (‘ActiCount’) were estimated. In addition, the total number of steps and the average cadence (steps/min) were provided for each classified walking bout. PA was characterized by various univariate metrics including the percentage of time being sedentary, the percentage of time spent walking, the number of steps normalized to the measurement duration (in hours), and the mean cadence of walking bouts of each day. The univariate metrics were computed based on the original and the smoothed PA time series.

### 2.4. Complexity Analysis

Complexity was introduced in order to analyze the variability of biological and physiological time series data [23,24]. The technique was subsequently adapted and applied to analyze ambulatory activity patterns [25]. Paraschiv-Ionescu et al. proposed complexity analysis on a multivariate PA pattern (‘barcode’) derived from wearable sensor data [10]. The ‘barcode’ is constructed for the analyzed period based on the classified activity category, the duration and the intensity. The entropy rate of the resulting multi-state ‘barcode’ represents complexity. In the analysis of the pilot data, an adapted ‘barcode’ was used, where the ‘ActiCount’ was modeled according to a validation study presented in [19]. Entropy rate of the ‘barcode’ was computed in terms of Lempel-Ziv complexity based on the method described in [26]. (Additional materials are presented in Appendix A.1). Complexity of the ‘barcode’ generated from both the original and the smoothed time series of PA were computed to analyze the influence of activity classification on the calculated complexity.

### 2.5. Statistical Analysis

For each participant, the univariate metrics and the complexity metric of PA were analyzed for each day. The average value of each metric over the one-week measurement before (Week0) and during (Week3) the pilot study was calculated. According to study [27], participants having more than two days’ sensor data during the one-week measurement, in both Week0 and Week3, were included for statistical analysis. The changes in PA metrics between Week0 and Week3 were computed. The change in CBMS score pre and post the pilot study was calculated. The primary analysis was to examine the correlations between the change in PA metrics and the change in CBMS score. Given the small sample size and ordinal data type (CBMS scores), spearman coefficient (ρ) was used to analyse the strength of correlation. In addition, a non-parametric effect size calculator, Cliff’s Delta, was applied to measure the degree of overlap between the distribution of various variables extracted pre- and during/post interventions [28]. Cliff’s Delta approaching 1 or −1 indicates absence of overlap, whereas 0 indicates overlap completely. Wilcoxon signed rank test was applied to examine the statistical significance of the change. Changes with a *p* value < 0.05 was considered statistically significant. The secondary analysis consisted of the impact of PA time series smoothing on the conventional metrics and the complexity metric. The analysis compared the aforementioned statistical outcomes before and after smoothing. Additional analyses on Week0 data compared the distributions of the length of sedentary and walking bouts before and after smoothing. The coefficients of variance (CV) of the daily complexity value of the original and the smoothed PA time series were compared.

## 3. Results

**Figure 2:**
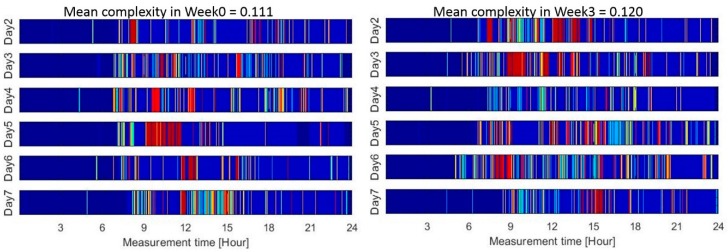
Barcode and mean complexity of one-week PA time series of Week0 (**left**) and Week3 (**right**) in one participant.

In total, 30 participants were included in the pilot study with 10 participants at each trial site. Due to technical problems with the sensor devices (no data could be retrieved), eight participants were excluded from further data processing and analysis (*n* = 5 in Week0 and *n* = 3 in Week3). One participant’s CBMS score was not available at the baseline assessment and was excluded from further data analysis leaving 21 participants for statistical analyses. All participants in statistical analysis had minimum 3 days of data in Week0 and Week3. Table 1 summarizes the descriptive statistics of PA metrics and CBMS score for the included participants (*n* = 21, except the CV of complexity, where *n* = 25 from Week0 were analyzed) in the pilot study. The included participants were not significantly different from the excluded participants in CMBS scores at the pre- (Willcoxon ranksum test, *p* = 0.14) or the post- (*p* = 0.23) intervention assessment. To illustrate, Figure 2 shows one participant’s barcode (based on smoothed PA time series) in Week0 (left) and Week3 (right). The barcode in Week3 shows richer colours filled in throughout several days of measurements, which was reflected by the higher mean complexity score of 0.120 compared to 0.111 in Week0.

**Figure 3:**
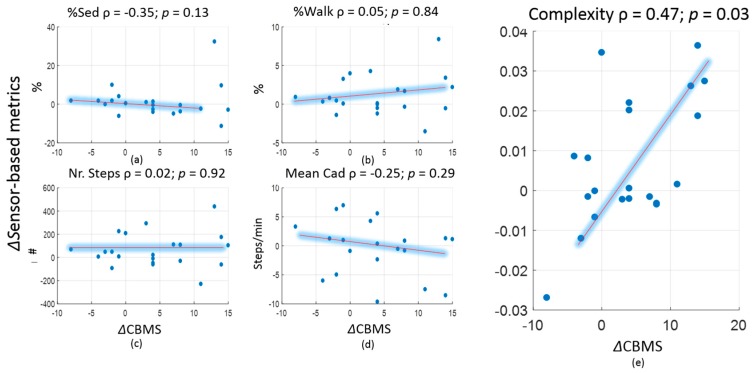
Spearman correlations between changes in PA metrics (based on original PA time series) and CBMS score. (**a**) Change in percentage of sedentary time vs. change in CBMS. (**b**) Change in percentage of walking time vs. change in CBMS. (**c**) Change in normalized number of steps vs. change in CBMS. (**d**) Change in mean cadence vs. change in CBMS. (**e**) Change in complexity vs. change in CBMS.

For the primary analysis, scatter plots in Figure 3 show the correlations. Spearman correlations between changes in PA metrics (based on original PA time series) and CBMS score (between the change in CBMS score and the changes measured by various conventional univariate metrics and the complexity metric) are based on original PA time series. Changes in univariate metrics had no significant association with the change as measured by the CBMS score. In contrast, complexity had a significant positive correlation (ρ = 0.47, *p* = 0.03) with the change in CBMS. Complexity was higher after intervention with an effect size of 0.18, which was comparable to the effect size as measured by the CBMS score (0.20). Despite an increase in complexity post intervention, the change was not statistically significant. Changes in univariate metrics had smaller effect size and were not statistically significant (see Table 1).

**Figure 4:**
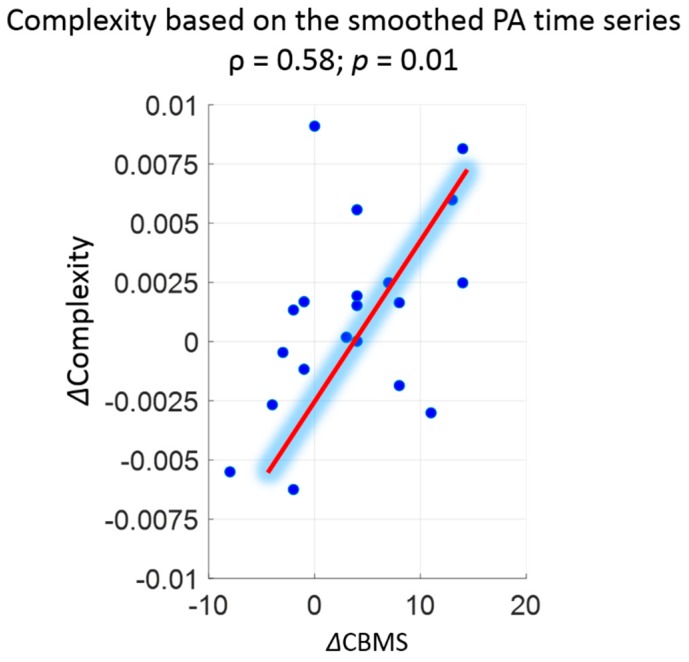
Association between complexity change and CBMS score change after smoothing PA time series.

For the secondary analysis, after smoothing the PA time series, multiple very short sedentary bouts were merged into one longer bout. Similarly, multiple walking bouts with short interruptions were concatenated to form a continuous walking bout (see Appendix A.2). Comparison of mean values of various univariate metrics presented in Table 1 indicated that smoothing had little impact on the percentage of sedentary time and the percentage of walking time, whereas the total number of steps and mean cadence were reduced. The value of complexity for the smoothed PA time series was smaller, compared to the original complexity. The change in complexity for the smoothed PA time series resulted in a stronger association with the change in CBMS score (ρ = 0.58, *p* = 0.01 as shown in Figure 4. Association between complexity change and CBMS score change after smoothing PA time series.). Moreover, the mean CV of complexity of the participants in Week0 decreased from 0.11 to 0.07 after smoothing the PA time series (see Appendix A.2).

## 4. Discussion

Authors should discuss the results and how they can be interpreted from the perspective of previous studies and of the working hypotheses. The findings and their implications should be discussed in the broadest context possible. Future research directions may also be highlighted. The primary analysis of this study focused on the relevance of various conventional PA metrics and the complexity in the assessment of functional change after an exercise intervention in younger older adults. In addition, we analyzed the impact of smoothing PA time series data on the calculation of various PA metrics and the complexity metric. Despite a very short intervention, the change in complexity was significantly correlated with the change as measured by the CBMS score, whereas, the changes in conventional PA metrics did not show significant correlation with the change as measured by the clinical assessment. Moreover, smoothing the PA time series, to aggregate short activity bouts, improved the complexity metric in terms of stronger correlation with functional change as measured by a clinical assessment and higher measurement reproducibility as quantified by the CV of one-week measurements.

These results revealed that complexity is a useful and a more sensitive metric than conventionally applied univariate PA metrics in the assessment of functional change in younger older adults. Conventional PA metrics derived from wearable sensors, step counts, or cadence, might not be sensitive enough to capture the functional change after short interventions. Metrics characterizing one aspect of daily physical activity, such as the time spent walking or being sedentary, do not provide a comprehensive picture of the determinants of functional status. The complexity metric of physical behaviour, on the other hand, characterizes the quantity, the quality, and the dynamic changes between different activities and different performances while doing the same activity (such as a change in cadence) in the ‘barcode’. Further, the entropy rate increases while the number of sub-patterns in the ‘barcode’ increases as illustrated in Figure 2. Since it captures more aspects of physical behaviour simultaneously, the complexity metric has the potential to capture the underlined important aspect in the aging process that the variety and dimension of activities decreases due to functional decline [29,30].

The ‘barcode’, as defined in Table A1 and illustrated in Figure 2, is constructed with generic activity features derived from the wearable sensors. This makes complexity a generic metric for PA data analysis in principle without constraints in specific sensor configuration or wearing position. The activity features required by the ‘barcode’, such as walking time and number of steps, are universally recognized by the state-of-the-art wearable sensors [10,12,25]. Selection of activity features to be included in barcode construction is a topic worth separate investigation. For example, efficacy and sensitivity to wearing position of a ‘barcode’ that states sensor-derived activity levels (sedentary, light, and moderate-to-vigorous) can be analyzed [31].

**Figure A1:**
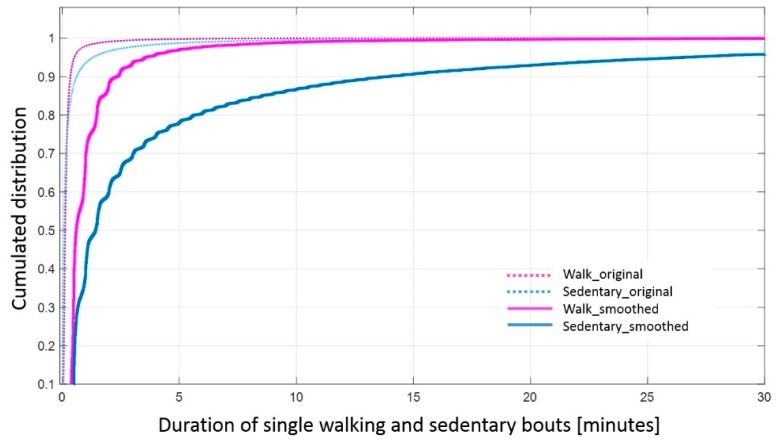
Comparison of cumulated distribution of walking and sedentary bouts before and after smoothing of activity classification output.

The complexity metric analyses the entropy rate of the ‘barcode’. Changes in ‘barcode’ states depend on the richness of the activity performed but is also influenced by the resolution of the activity features. The higher the resolution, the more detailed the features in the activity performed can be described; however, the resolution becomes less resistant to noise in the activity data. As demonstrated in Figure 1, the original PA time series (the top bar) has second-by-second feature resolution, which shows frequent fast changes between sedentary and walking activities (for example, see between 1000 and 1250 s). In the original PA time series, almost all sedentary bouts were shorter than five minutes, and less than 5% of walking continued for more than one minute in all participants (see distribution of PA activity data before and after smoothing in Figure A1). It is plausible that very short bouts were artifacts of the activity features due to the noise in the accelerometry signal acquired at the waist. The complexity metric aims to capture the dynamic change of real activity patterns that are encoded in the ‘barcode’ rather than the signal noise. Thus, a pre-processing method to remove the noise, or smooth the activity features before barcoding for complexity analysis is necessary and important. We proposed a smoothing method in this study to remove the noise. As shown in the secondary analysis, the resulting complexity value was lower than the complexity of the original PA time series, indicating that there was less frequent change in the activities after smoothing. The smoothing method improved the reproducibility of the complexity metric, which implies that the smoothing removed irrelevant noise existing in the original PA time series. However, it preserved the clinically relevant activity patterns as shown in the significant correlation with the clinical outcome. We observed a decrease in step counts and mean cadence after smoothing, which was likely due to the procedure, where steps in very short walking bouts were removed, but no step was inserted in the concatenated walking bouts.

There are some limitations in the study presented in this manuscript. First, the sample size was relatively small. Due to missing data, only 21 participants were included in the statistical analyses. Even though, there was no significant difference in functional status as measured by the CBMS score between the included and the excluded subject, the strength of association and effect size of change measured in this pilot study will need to be confirmed in a larger cohort of comparable participants. Secondly, the assessment of daily activity with wearable sensors during the intervention (Week3) was collected one week prior to the post-intervention assessment of CBMS. For the short intervention pilot, this time discrepancy might have introduced bias. This bias likely has made our estimates of pre- or post-intervention change in PA metrics to be more conservative. Lastly, the window of 30 s chosen for the smoothing method is based on the lowest threshold for walk analysis in the ‘barcode’ design. Conceptually, this threshold corresponds to most indoor walking activities [7,10]. However, future research should conduct a systematic evaluation to confirm the optimized activity feature resolution for complexity analysis.

In the context of PreventIT project, the on-going multi-national randomized controlled trial (RCT) study will provide a larger cohort data of 180 participants with comparable demographic and health profile as studied in this pilot. The RCT will assess the participant’s CBMSs and monitor their PAs using a similar sensor configuration at baseline, 6-month, and 12-month follow-up. Correlation between the change in complexity and the change in CBMS will be validated after the longer intervention. Analyses on the association between complexity and CBMS at baseline will be conducted. In addition, relationship between complexity and each individual balance and mobility components assessed in CBMS will be analyzed.

## 5. Conclusions

This study demonstrated the clinical relevance of using a multivariate metric for physical behavioural complexity to capture change in functional status in a longitudinal study in younger older adults. The complexity metric showed higher sensitivity to functional change than conventionally applied univariate PA metrics such as sedentary time and step count. Complexity can be applied as a generic metric to analyze the daily life activity patterns derived from wearable sensors. A meaningful resolution of sensor-derived activity features is important for reliable complexity analysis. The complexity metric is a useful metric to be further developed for the outcome measure of the feasibility and the effectiveness of PreventIT interventions.

## Figures and Tables

**Table 1:** Descriptive statistics of PA metrics, complexity, and CBMS scores in pre- and post- intervention assessments. PA metrics and complexity based on original and smoothed PA time series are compared with CMBS scores.

	Week0 or Pre Pilot (Mean ± SD) Original/Smoothed	Week3 or Post Pilot (Mean ± SD) Original/Smoothed	Association (ρ) with CBMS Original/Smoothed	Effect Size (Cliff’s Delta) Original/Smoothed
**Percentage of sedentary time (%)**	44.9 ± 6.0/47.7 ± 6.5	44.4 ± 5.6/47.5 ± 6.0	−0.35/−0.28	−0.12/−0.08
**Percentage of walking time (%)**	9.1 ± 2.0/9.1 ± 2.2	9.9 ± 3.0/10.0 ± 3.3	0.05/−0.01	0.13/0.15 ^a^
**Normalised nr. of steps (steps/hour)**	489 ± 123/361 ± 111	532 ± 182/395 ± 160	0.02/−0.17	0.11/0.08
**Mean cadence (steps/minute)**	78 ± 5/52 ± 8	78 ± 6/51 ± 7	−0.25/−0.33	0/−0.13
**Complexity**	0.178 ± 0.024/0.101 ± 0.006	0.185 ± 0.024/0.103 ± 0.007	0.47 ^a^/0.58 ^a^	0.18/0.15
**CV of complexity ^b^**	0.11 ± 0.05/0.07 ± 0.02			
**CBMS score**	66.4 ± 12.8	70.2 ± 12.9		0.20 ^a^

^a^ Statistically significant (*p* < 0.05), ^b^ based on 25 participants’ data at Week0.

**Figure A2:**
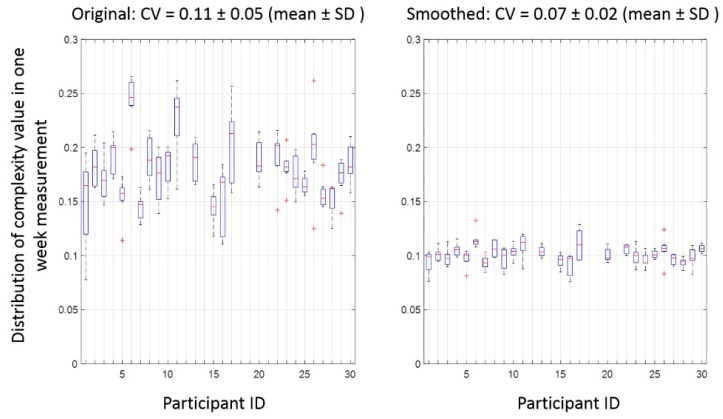
CV before and after smoothing PA time series.

## Appendix A

### Appendix A.1. Adapted Barcode Design and Complexity Computation

A ‘barcode’ state was assigned to each second of the time series according to Table A1. The definition was modified for the type ‘active’ from the original design presented in [10] that applied the absolute value of acceleration. As acceleration is sensitive to the sensor wearing location, the acceleration based threshold was replaced by the ‘ActiCount’ in this study. The ‘ActiCount’ thresholds are determined based on a validation study presented in [19]. Complexity computation was according to Equation (A1), where ‘nrPattern’ is the total number of sub-patterns found in the ‘barcode’. ‘nBC’ is the total number of ‘barcode’ states. In our analysis, nBC = 18. N is the total length of PA time series in seconds.

(A1) $$\mathrm{Complexity}=\frac{\mathrm{nrPattern}*\left(\frac{\log_{10}\left(\mathrm{nrPattern}\right)}{\log_{10}\left(\mathrm{nBC}\right)}+1\right)}{N}$$

**Table A1:** Definition of barcode state according to PA category, intensity and duration.

Category	Intensity	Duration	State
Lying			1
Sedentary			2
Active	ActiCounts ≤ 3500 (counts/minute)		3
	3500 < ActiCounts ≤ 7000		4
	7000 < ActiCounts ≤ 10000		5
	ActiCounts > 10000		6
Walking	Cadence ≤ 60 (steps/minute)	Duration ≤ 30 s	7
	60 < Cadence ≤ 90		8
	90 < Cadence ≤ 140		9
	Cadence > 140		10
	Cadence ≤ 60	30 < Duration ≤ 120	11
	60 < Cadence ≤ 90		12
	90 < Cadence ≤ 140		13
	Cadence > 140		14
	Cadence ≤ 60	Duration > 120	15
	60 < Cadence ≤ 90		16
	90 < Cadence ≤ 140		17
	Cadence > 140		18

### Appendix A.2. Effect of Smoothing on the Duration of Activity Bouts and Reliability of Complexity

Figure A1 shows that, after smoothing, 90% of the sedentary bouts lasted up to 15 min, whereas only 10% of the walking bouts were longer than two and half minutes before a stop. Figure A2 compares the mean and standard deviation of daily complexity in one-week measurement before and after smoothing. Smoothing lowered the mean values and the CV of the complexity.
